# Radiological, Pathological, and Surgical Outcomes with Neoadjuvant Cemiplimab for Stage II–IV Cutaneous Squamous Cell Carcinoma in the Deep Sequencing in Cutaneous Squamous Cell Carcinomas (DISCERN) Trial

**DOI:** 10.3390/cancers17101727

**Published:** 2025-05-21

**Authors:** Annette M. Lim, Benjamin Baker, Peter Lion, Christopher M. Angel, Jennifer Simmons, Bryce Jackson, Matthew Magarey, Angela Webb, Kevin Nguyen, Jo Hudson, Kwang Yang Chin, Anthony Cardin, Rajeev Ravi, Edwin Morrison, Tam Quinn, Ian Hunt, Danny Rischin

**Affiliations:** 1 Department of Medical Oncology, Peter MacCallum Cancer Centre, Melbourne, VIC 3000, Australia; jo.hudson@petermac.org (J.H.); danny.rischin@petermac.org (D.R.); 2 Sir Peter MacCallum Department of Oncology, University of Melbourne, Parkville, VIC 3010, Australia; 3 Department of Plastic Surgery, Manchester University NHS Foundation Trust, Manchester M13 9WL, UK; benjamin.baker@nhs.net; 4 Department of Head & Neck Surgery, University College London Hospitals NHS Foundation Trust, London NW1 2PG, UK; peter.lion@nhs.net; 5 Department of Pathology, Peter MacCallum Cancer Centre, Melbourne, VIC 3000, Australia; christopher.angel@petermac.org; 6 Parkville Cancer Clinical Trials Unit, Peter MacCallum Cancer Centre, Melbourne, VIC 3000, Australia; jenny.simmons@petermac.org; 7 Complex Skin Unit, Department of Plastic Surgery, Peter MacCallum Cancer Centre, Melbourne, VIC 3000, Australia; bryce.jackson@petermac.org (B.J.); angela.webb@petermac.org (A.W.); edwin.morrison@petermac.org (E.M.); tam.quinn@petermac.org (T.Q.); 8 Department of Head and Neck Surgery, Peter MacCallum Cancer Centre, Melbourne, VIC 3000, Australia; matthew.magarey@petermac.org (M.M.); kevin.nguyen@petermac.org (K.N.); 9 Department of Cancer Imaging, Peter MacCallum Cancer Centre, Melbourne, VIC 3000, Australia; kwang.chin@petermac.org (K.Y.C.); anthony.cardin@petermac.org (A.C.); rajeev.ravi@petermac.org (R.R.); 10 Biomathematics and Statistics Scotland, The James Hutton Institute, Invergowrie, Dundee DD2 5DA, UK; ian.hunt@bioss.ac.uk

**Keywords:** neoadjuvant, immunotherapy, cemiplimab, cutaneous squamous cell carcinoma, CSCC, iPERCIST, pathology, surgery, plastic, radiology, skin cancer

## Abstract

**Background:** A previous published Phase 2 trial using 2–4 doses of neoadjuvant cemiplimab in stage II–IV resectable cutaneous squamous cell carcinoma (CSCC) demonstrated that a complete pathological (pCR) rate of 51% and major pathological response (mPR) rate of 13% could be achieved with durable disease control. **Methods:** In this open-label, single-institution phase II trial (NCT05878288), patients with stage II–IV resectable CSCC received up to four doses of neoadjuvant cemiplimab prior to surgery. The primary endpoint of the study was to perform comprehensive molecular profiling. The focus of this report are the secondary clinical endpoints of pCR rate, mPR (defined as <10% viable tumour) rate, overall response rate (ORR) using Response Evaluation Criteria in Solid Tumours (RECIST) 1.1, immune-modified RECIST (imRECIST) and Immune PET Response Criteria in Solid Tumours (iPERCIST), disease-free survival (DFS), overall survival (OS), safety, and to describe changes in planned surgery. **Results:** Eleven patients were enrolled, with all proceeding with surgery. An ORR and pCR rate of 73% (8/11; 95% CI 0.39–0.93) was achieved, whilst 3/11 patients progressed on treatment. On pre-operative imaging, all 8/11 pCR patients demonstrated a partial response (RECIST 1.1), whilst 6/8 achieved a complete metabolic response and 2/8 a partial metabolic response (iPERCIST). Median follow-up was 10.2 (IQR 6.7–16.4) months. DFS was 91% (95% CI 0.57–1) and OS was 100% (95% CI 0.68–1), with one non-responder patient who developed recurrent locoregional and distant metastatic disease. There were no unexpected safety signals. Pathological features of response to neoadjuvant immunotherapy most commonly were granulomatous inflammation with keratin, fibrosis and inflammation. No cases with a dense inflammatory infiltrate were observed. Neoadjuvant immunotherapy did not impact the intra-operative planning and execution of surgery, but in the eight pCR cases, it reduced the extent of required surgery, whilst in the three non-responder cases, surgery was more extensive than originally planned. **Conclusions:** The DISCERN trial confirms that an excellent complete response rate can be achieved with four doses of neoadjuvant immunotherapy in stage II–IV CSCC. Proposed refinements to the pathological assessment of response and metabolic response criteria in CSCC for the neoadjuvant context are provided.

## Simple Summary

The DISCERN trial confirms that an excellent complete pathological response rate of 73% can be achieved with the use of four cycles of neoadjuvant cemiplimab prior to surgery for patients with stage II–IV cutaneous squamous cell carcinoma (CSCC), highlighting the importance of a longer duration of dosing. Given that a prior phase 2 trial reported significant discordance in 10/70 (14%) of cases between local and central pathological review, the histopathological response features of CSCC to neoadjuvant immunotherapy are described in detail, with criteria proposed to account for the presence of anucleate keratin and cavitation to standardize the measurement of tumour bed dimensions for the calculation of residual viable tumour. Correlates between radiological response assessments (RECIST 1.1, imRECIST and iPERCIST) and pathological outcome are reported, with refinement of iPERCIST criteria in the neoadjuvant context proposed to also include resolution of avidity symmetrical to a contralateral organ without disease (i.e., physiological avidity).

## 1. Introduction

Cutaneous squamous cell carcinoma (CSCC), arising from epidermal keratinocytes, contributes to Australasia having the highest reported incidence worldwide of non-melanomatous skin cancer (NMSC), with ~756,400 (95% UI) diagnosed in 2019 [1,2,3]. Globally, CSCC deaths are now comparable to melanoma [4]. The true burden of disease is likely underestimated as most cancer registries worldwide do not formally capture the incidence of CSCC, and because many patients present with multiple synchronous, metachronous, and recurrent NMSC [5]. Although most CSCC is curable with surgery and/or radiotherapy, both the treatment and disease can lead to significant functional and aesthetic morbidity that can have a profound impact on quality of life. Due to the tendency of CSCC to develop on sun-exposed sites such as the head and neck, surgery can require orbital exenteration or resection of other anatomical units such as the nose, cheek, lip, ear, or scalp [6,7,8,9].

The treatment paradigm for recurrent or metastatic CSCC not amenable to surgery or radiation therapy has been transformed with immune checkpoint blockade (ICB) specifically targeting the Programmed Death-1 (PD-1) receptor and ligand (PD-L1) axis, which achieves an objective response rate of ~50%, with durable control and rapid improvement of quality of life [10,11,12,13,14]. These findings and an increasing understanding of the value of neoadjuvant ICB prior to surgery in other tumour types have led to exploration of the use of immunotherapy in the neoadjuvant setting for CSCC [15,16,17]. A multi-centre phase II trial (NCT04154943) used 2–4 cycles of neoadjuvant cemiplimab for the treatment of 79 patients with stage II–IV CSCC, and achieved a complete pathological response (pCR) in 51% (95% confidence interval [CI], 39–62) and a major pathological response (mPR) in 13% (95% CI, 6–22) of patient tumours [18]. Recent follow-up data pooled from 51 patients on the pilot and phase 2 studies demonstrated that no disease recurrences have been experienced in any patients who achieved a pCR at surgery, and of those who completed surgery, 12-month disease-free survival was 92% (95% CI 82–97) [19].

Given that some tumours are refractory to immunotherapy, it is critical to be able to predict which patients will benefit in order to optimise patient selection for neoadjuvant ICB approaches and to understand the biological mechanisms underpinning response and resistance. To address this need, the Deep sequencIng in cutaneous Squamous Cell caRciNomas (DISCERN) trial was designed with a primary objective to comprehensively profile the tumour molecular ecosystem of CSCC from patients treated with 2–4 doses of neoadjuvant cemiplimab using single-cell sequencing and bulk genomic profiling. The primary objective will be reported subsequently, when analysis has been completed. Here, we report the secondary objectives of the DISCERN trial, including the imaging, pathological, and surgical results, which highlight important considerations in these areas for upcoming phase 3 trials.

## 2. Materials and Methods

### 2.1. Study Oversight

The trial was an investigator-initiated trial, and the study sponsor was the Peter MacCallum Cancer Centre. The trial commenced on 23 May 2023 and was conducted in accordance with the Declaration of Helsinki [20] and International Conference on Harmonization Good Clinical Practice Guidelines [21]. Local institutional ethics committee approval was obtained (22/124) prior to study commencement, and all recruited patients provided written informed consent prior to study participation.

### 2.2. Study Population

In brief, eligible patients were aged 18 years or over; had a histologically confirmed diagnosis of stage II–IV (M0) CSCC (for head and neck subsites, staging was based on the 8th edition of the American Joint Committee on Cancer Staging Manual [22], and for other subsites, staging was based on the 9th edition of the Union for International Cancer Control Manual of Clinical Oncology [23]) with at least one measurable lesion as per the Response Evaluation Criteria in Solid Tumours (RECIST) version 1.1 criteria [24]; with an Eastern Cooperative Oncology Group (ECOG) performance status score of 0 or 1; with an anticipated life expectancy of greater than 12 weeks; with adequate end organ hepatic, renal, and bone marrow function; and who consented to trial mandated research biopsies and blood tests taken at baseline, during treatment, and at surgery.

Exclusion criteria included the presence of active other solid malignancy or haematological malignancies (unless considered indolent or non-life threatening); presence of distant metastatic disease; active autoimmune disease or interstitial lung disease or pneumonitis requiring systemic therapy within the last 5 years; steroid use >10 mg prednisolone within 14 days of study drug (except physiological replacement or for hypersensitivity); active infection requiring treatment including human immunodeficiency virus (HIV)-1 or HIV-2 serum antibody, hepatitis B virus, or hepatitis C virus, or active tuberculosis; breast feeding or positive pregnancy test; receipt of live vaccine within 30 days of the first study treatment; prior transplant receipt; prior PD-1/PD-L1 inhibitor exposure for the same lesion as enrolment; true squamous cell carcinoma of unknown primary site; any anticancer treatment other than radiation therapy within 30 days of initial administration of cemiplimab or planned to occur during the study period; history of allergy or hypersensitivity to antibody treatments or to cemiplimab or its excipients; institutionalised patients; and patients not willing to comply with mandated study procedures.

### 2.3. Study Design, Treatment and Procedures

The DISCERN trial was a prospective, single-centre, open-label study that recruited patients with stage II–IV (M0) CSCC who were candidates for curative surgery to receive 2–4 doses of neoadjuvant cemiplimab.

After screening over a 28-day period, eligible and consented participants received up to 4 doses of neoadjuvant cemiplimab 350 mg at 3-weekly intervals for up to 12 weeks prior to surgery, or until unacceptable toxicity, disease progression, or withdrawal of consent. Patients were reviewed prior to each dose and underwent tumour response imaging assessment with fluorodeoxyglucose-positron emission tomography (FDG-PET)/diagnostic computerised tomography (CT) +/− magnetic resonance imaging (MRI) scans as required at baseline, prior to the third dose of cemiplimab (~day 43) and prior to surgery (~day 85). Toxicity is monitored from treatment start until the end of follow-up and graded according to the National Cancer Institute Common Terminology Criteria v5.0 [25]. Patients had fresh tissue biopsies taken with biomarker study bloods at baseline, prior to the second dose of cemiplimab (~day 22) and at surgery. All patient management was discussed at multidisciplinary team meetings, and if a patient met criteria to discontinue, the treating clinicians could consider early surgical intervention. Surgery and pathological response assessment were performed as per standard of care. Completion of surgery defined the completion of study treatment. Adjuvant therapy was considered part of the standard of care. Follow-up is performed every 6 months for 24 months after the completion of surgery, with clinician review for adverse events (AE), concomitant medications, FDG-PET/diagnostic CT imaging assessments, and research blood analyses.

#### 2.3.1. Radiological Assessments

Objective response rates (ORRs) were investigator-assessed using expert radiologist measurements and multidisciplinary meeting review, using both RECIST 1.1 [24] and immune-modified RECIST (imRECIST) [26]. Confirmation of complete or partial response per imRECIST was not required or possible given the short duration of the neoadjuvant intervention. FDG-PET scans were assessed by a nuclear medicine specialist/radiologist (A.C.) according to the immune PET Response Criteria in Solid Tumours (iPERCIST) [27,28], with both standardised uptake value peak (SULpeak) and maximum standardized uptake value (SUVmax) measurements calculated. Only SULpeak measurements are reported per iPERCIST criteria.

#### 2.3.2. Pathological Assessments

Surgical samples were examined per standard of care by an expert pathologist, which informed the multidisciplinary team’s decision regarding the need for adjuvant therapy. The entire excised tumour bed and/or involved nodes were submitted for serial sectioning and pathological assessment of response. Study-related pathology reporting included assessment of the tumour resection margins (distance of margins from viable disease); the tumour bed/regression bed resection margins (distance of the margins from areas which previously had evidence of disease but no viable tumour was present after therapy); largest measurement of areas with viable tumour; largest measurement of the tumour bed/regression bed size; presence of lymphovascular and perineural invasion; presence of extranodal extension; a description of the predominant pattern of regression if present—as inflamed, necrotic, fibrotic, mixed, or granulomatous; and assessment of the density (dense 3+, moderate 2+, minimal 1+) and presence of pathological features of immunotherapy response, which included the presence of tertiary lymphoid structures, plasma cells, cholesterol clefts, foamy macrophages, neovascularisation, proliferative fibrosis, granulomas, giant cells, and immune exclusion. Similar to previous reports [15,29,30,31], pathologists would quantify the percentage residual viable tumour (RVT) as a measurement of the surface area of the residual tumour divided by the surface area of the tumour bed for both the primary and/or involved nodes, aggregating all involved areas. After each standard-of-care reporting pathologist completed their assessment, all cases and study pathology reports were reviewed and finally annotated by an expert head and neck pathologist (C.M.A.) to ensure consistency.

#### 2.3.3. Surgical Assessments

Prior to the commencement of treatment, the surgical team were asked to document the planned surgery that would occur if neoadjuvant immunotherapy was not available. At the time of surgery, the surgical team was asked to document on a provided template the surgical planning and intraoperative findings for each case contemporaneously. Surgical descriptions included expectations about residual disease based on macroscopic assessment; planned margins and extent of surgery for nodal disease and how these were determined; and the impact of neoadjuvant treatment on tissue feel, surgical planes, and surgical technique.

### 2.4. Endpoints

The primary endpoint of the trial was the successful execution and generation of data from single-cell sequencing and bulk genomic profiling of CSCC from patients treated with neoadjuvant immunotherapy, without a pre-specified threshold number of successful samples required, given the exploratory nature of the study.

Clinical secondary endpoints were to evaluate pCR rate (defined as the proportion of cases with no viable tumour identified), mPR rate (defined as the proportion of tumours with <10% viable tumour), and overall response rate (ORR) using imaging assessment RECIST 1.1 [24], imRECIST [26], and iPERCIST response criteria [27,28]; to compare tumour response assessed by pathology and imaging; to report on disease-free survival (DFS) and overall survival (OS); to describe immune-related adverse events (irAE) ≥ grade 2, AEs ≥ grade 3 and serious adverse events (SAEs); and to describe any changes in the extent of surgical plans with the use of neoadjuvant immunotherapy.

Translational secondary endpoints were to describe the molecular differences between immunotherapy responders versus non-responders, and to describe the extent to which tumour microenvironment characteristics were detected in whole blood and how these corresponded to treatment responses.

### 2.5. Statistical Analyses

Study data were collected and managed using the Research Electronic Data Capture (REDCap) tools hosted at the Peter MacCallum Cancer Centre [32,33].

As the primary objective of the study was translational and descriptive, a formal power calculation based on the pragmatic sample size was not performed. The initial sample size of 10 participants was increased to 11 after one participant’s baseline single-cell sample did not pass the initial quality assurance assessment in terms of the expected number of single cells analysed.

The analysis sample set consisted of all enrolled participants with one tumour sample collected and who received at least one dose of cemiplimab. Conventional summary and descriptive statistics were used for continuous variables, including (as appropriate) means, quantiles (including medians), standard deviations, and 95% confidence intervals (CIs). Categorical data is summarised (as appropriate) with counts, proportions, and 95% CIs for the proportions. CIs for proportions use Wilson’s method with a continuity correction. All statistical analysis was performed with R (R Core Team 2024, version 4.1). DFS was defined as the time from the date of surgery to the date of first disease recurrence or death due to any cause, whichever occurred first. OS was defined as the time from the first dose of cemiplimab to the date of death due to any cause. Deaths and other SAEs will be listed and described. Disease progression and death due to disease were not considered as SAEs or AEs.

## 3. Results

### 3.1. Patient Disposition and Characteristics

A total of 11 patients were enrolled on the DISCERN trial between 10 July 2023, and 11 July 2024. There was one screen failure due to hypercalcemia of malignancy impacting expected life expectancy (see Supplementary Figure S1 for the Consolidated Standards of Reporting Trials flow diagram). At the time of data cut-off (12 December 2024), the median follow-up was 10.3 (interquartile range 6.7–16.4) months. Baseline characteristics of the patients are summarised in Table 1.

The median age of participants was 67 (range 55–83) years, and 91% were male, with the majority having an ECOG performance status of 0 (8/11, 73%). The majority (9/11, 82%) had CSCC of the head and neck as the primary tumour site, and 8/11 (73%) had stage IV (M0) disease. A total of 8 (8/11) participants received four doses of neoadjuvant cemiplimab; 3 (3/11) patients were diverted to surgery early due to disease progression; 1 patient went to surgery after one dose of cemiplimab due to the development of a through-and-through mucosal defect of a CSCC of the lower lip with clinical and radiological progression; 1 patient (who was initially thought to have possible pseudoprogression during an admission for an intercurrent infection) went to surgery after two doses of cemiplimab; and 1 patient went to surgery after three doses of cemiplimab following biopsy confirmation of new non-RECIST measurable nodal disease that was identified on imaging at Day 43 despite a partial response (−45%) in the target axillary nodal lesion and clinical response in the primary hand lesion (non-target lesion, NTL). All patients who achieved a pCR with neoadjuvant therapy were not recommended to receive adjuvant treatment by multidisciplinary team consensus, whilst all pathological non-responder (pNR) patients were recommended adjuvant radiation therapy.

### 3.2. Clinical Efficacy

All 11 participants received surgery as per the protocol. A pCR was observed in 8/11 (73%; 95% CI, 0.39–0.93) patients, whilst the 3/11 (27%) patients with clinical and radiological progressive disease had >50% residual viable tumour or new disease (Table 2 and Table 3). Using imaging response assessment, the 8/11 patients who achieved a pCR had a partial response by RECIST 1.1 (73%; 95% CI 0.39–0.93), and the remaining 3/11 (27%) pNR had progressive disease. Response assessments per imRECIST and iPERCIST are detailed in Table 3, with imRECIST outcomes similar to RECIST 1.1. Utilising iPERCIST metabolic response assessments, of the 8/11 (73%) patients with a pCR, only 2/8 achieved a complete metabolic response (CMR) on FDG-PET imaging prior to surgery, whilst 6/8 achieved a partial metabolic response (PMR). Representative examples of radiological response and progressive disease are demonstrated in the Supplementary Figures S2 and S3.

The median DFS for all patients was 91% (95% CI 0.57–1), with an OS of 100% (95% CI 0.68–1). One pNR participant with new level V cervical nodal disease developed recurrent locoregional and metastatic disease, detected with CT imaging prior to the commencement of adjuvant radiation.

### 3.3. Safety

The tolerability of neoadjuvant cemiplimab was similar to previous reports [11,18,34], with all adverse events and cemiplimab-related adverse events summarised in Supplementary Tables S1 and S2. There were five SAEs, with one treatment-related SAE of Grade 3 immune-related hypophysitis that developed post-operatively in a patient 136 days after the last dose of immunotherapy, who was treated with physiological steroid replacement. The four SAEs not related to treatment included one patient with grade 3 hyperkalaemia requiring hospitalisation secondary to worsening of chronic renal failure in the context of commencement of new anti-hypertensive medications, and one patient who had three separate hospitalisations for a fall, urinary tract infection, and an episode of confusion. The most common cemiplimab-related adverse events included fatigue (3/11, 27%) and maculopapular rash (3/11, 27%), but were grade limited to grade 1 and 2 events. No adverse events led to treatment delays or discontinuation. There have been no deaths in the study, and surgery was not delayed for any participant.

### 3.4. Pathological Description of Neoadjuvant Response

The pathological assessment of surgical specimens from the 8/11 patients with pCR and 3/11 patients with pNR disease is summarised in Table 4. In the 8/11 patient samples with no evidence of RVT, it was noted that the regression bed involved the resection margins in most cases or was microscopically close, and evidence of regressed extra-nodal extension was also observed in two cases. Of the three pNR cases, two patients had a mixed pathological response observed in different anatomical locations of the disease. For example, following three doses of neoadjuvant immunotherapy, one participant (011) with a primary hand lesion and axillary nodal disease at baseline had 15% RVT in the primary lesion, >50% RVT in the axillary nodal disease, but 100% RVT in new level V cervical nodal disease, consistent with clonal evolution.

**Figure 1:**
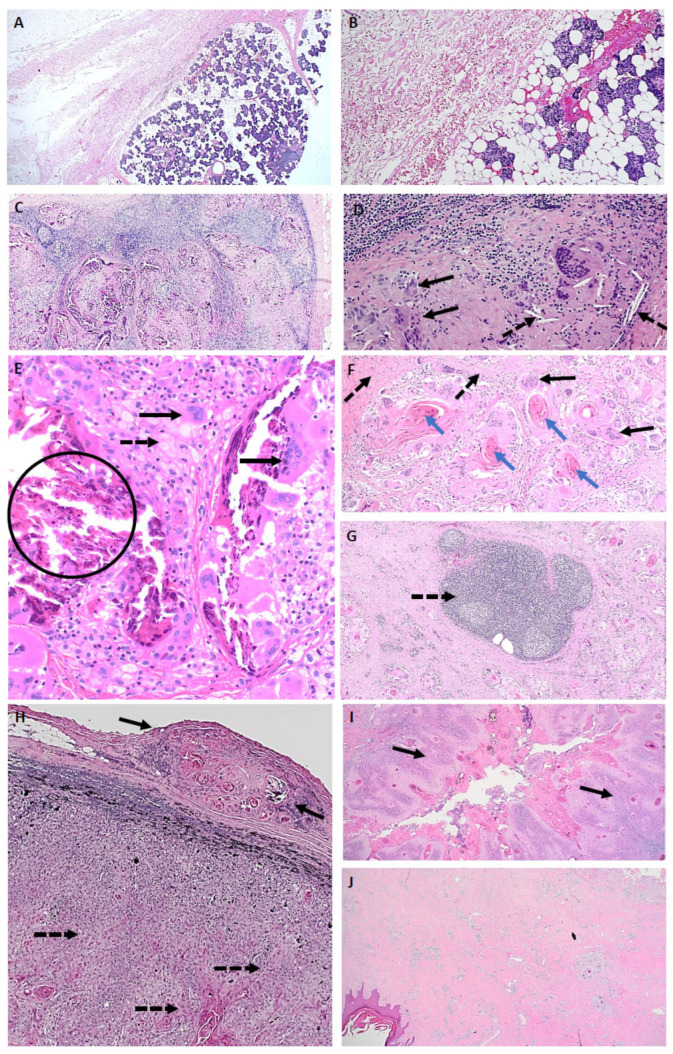
Pathological features of a complete response to neoadjuvant immunotherapy. (**A**) ID005—Normal: parotidectomy specimen with complete pathological response, normal tissue appearance and architecture, and absence of a detectable tumour bed or an inflammatory response (Mag ×20). (**B**) ID005—Normal: complete pathological response with normal tissue appearance and absence of an inflammatory response (Mag ×100). (**C**) ID002—Mixed regression pattern: lymph node includes granulomatous inflammation, fibrosis, keratin, residual lymph node structure, and no residual viable tumour (Mag ×40). (**D**) ID002—Mixed regression pattern: lymph node with cholesterol clefts (dotted arrow), giant cells (solid arrow), and no residual viable tumour (Mag ×200). (**E**) ID002—Mixed regression pattern: foamy macrophages (dotted arrow), giant cells (solid arrows), calcification (encircled), and no residual viable tumour (Mag ×200). (**F**) ID001—Fibrotic regression pattern with granulomatous reaction to keratin: fibrosis (dotted arrows), giant cells (solid arrows) associated with keratin (blue arrows), and no residual viable tumour (Mag ×40). (**G**) ID001—Fibrotic regression pattern: tertiary lymphoid structure (dotted arrow) observed in a specimen with a dominant fibrotic regression pattern and no residual viable disease (Mag ×40). Features of a pathological non-responder with a differential response to neoadjuvant immunotherapy in nodal metastases and the primary skin lesion from the same patient. (**H**) ID011—lymph node with evidence of tumour regression in the extracapsular extension of disease (solid arrows), whilst multiple areas of infiltrating residual viable tumour are observed within the lymph node (dotted arrows) (Mag ×40). (**I**) ID011—Section of the primary hand cutaneous squamous cell carcinoma, demonstrating areas of viable tumour (solid arrows) with no regression (Mag ×20). (**J**) ID011—Section of the primary hand cutaneous squamous cell carcinoma demonstrating areas of tumour regression (largely fibrosis) without evidence of viable cells (Mag ×20).

Specific pathological features following neoadjuvant immunotherapy are summarised in Table 5, which demonstrates that in the 8/11 pCR patients, there were different dominant response patterns observed, including one participant’s samples (005) that had no evidence of regression but completely normal histological architecture and appearance (Figure 1). In 4/8 pCR cases, the presence of granulomatous inflammation with fibrosis and keratin was observed (Figure 1), but this pattern was also noted in one pNR case that had a mixed clinical response in different anatomical locations. The only consistent pathological features observed in the majority of the pCR cases were the presence of granulomas and giant cells with variable density, which was similarly observed in the two pNR cases with a mixed response at different anatomical sites of disease. There was a variable presence of other pathological features of neoadjuvant response to immunotherapy, including the presence of tertiary lymphoid structures, neovascularisation, and cholesterol clefts, and no case had a dense infiltration of tumour-infiltrating lymphocytes (Figure 1).

### 3.5. Surgical Assessments

The receipt of neoadjuvant cemiplimab reduced the extent of surgery received in all patients who were found to have a pCR, given that clinical examinations during treatment reflected the partial responses identified on pre-operative imaging (Table 6). One participant (006) who had a large lower lip CSCC that impaired speech, mastication, and closure of his mouth had been planned to receive a total lower lip resection with free flap reconstruction and bilateral levels I–III neck dissections and post-operative radiotherapy. Following a complete clinical response to four neoadjuvant doses of cemiplimab and partial response on imaging assessments, the actual surgery performed after multidisciplinary team consensus was a wedge resection of the lip with a selective neck dissection of only level Ia and bilateral Ib nodes to avoid functional and aesthetic morbidity. Conversely, for those patients who progressed on immunotherapy, surgery was more extensive than originally planned, given that, in general, lesions were larger or more extensive than at baseline.

A summary of the intra-operative surgical considerations is provided in Supplementary Table S3, whilst noting that different surgical procedures were required according to the different anatomical locations of disease. In general, beyond the changes in planned surgery described above, neoadjuvant immunotherapy did not impact surgical execution regardless of whether the CSCC had responded to immunotherapy or not. Intra-operatively, it was difficult to predict clinically and by palpation whether residual disease was present or not. Neoadjuvant immunotherapy did not significantly impact the feel of tissues, although in the pCR cases, it was notable that fibrosis was predominantly felt by the surgeon in nodal disease rather than in a primary skin lesion, if present.

## 4. Discussion

The use of neoadjuvant immunotherapy in tumours susceptible to immune checkpoint blockade, such as CSCC, has the clear potential to alter the therapeutic paradigm to improve patient outcomes whilst avoiding or minimising the significant morbidity associated with multimodality treatment and adjuvant treatment approaches [15,16,17,18,19]. The DISCERN trial, which used four doses of neoadjuvant cemiplimab prior to surgery, has confirmed that an excellent pathological complete response rate (8/11, 73%; 95% CI 0.39–0.93) can be achieved in resectable stage II–IV CSCC. Although limited by small numbers, this is the highest pCR rate reported to date in CSCC, with all participants proceeding with planned surgery. The original pilot study (n = 20) and first neoadjuvant cemiplimab phase II trial (n = 79) reported similarly high pCR rates of ~51% (95% CI, 39–62) and a mPR in ~13% (95% CI, 6–22) of patient tumours [15,18]. The lower pCR rates are mostly explained by the delivery of fewer cycles of neoadjuvant therapy prior to surgery, with the pilot trial administering only two doses of ICB, and 17/79 (22%) of participants in the phase II trial received less than four doses. Secondly, there was a reduced number of samples evaluable for pathological response in the phase 2 trial, given that 9/79 (11%) of participants did not undergo surgery. The De-Squamate trial reported as an abstract, administered “at least two doses” of neoadjuvant pembrolizumab in 27 patients with stage II–IV resectable CSCC, and observed a pCR rate of 15% and complete clinical response (defined as a CMR on FDG-PET plus mapping biopsies negative of residual CSCC) of 45% [35]. A recently published phase II trial using three doses of neoadjuvant atezolizumab (a PD-L1 inhibitor) in stage III–IV resectable CSCC reported a lower pCR rate of 35% (7/20; 95% CI, 15.4–59.2) and mPR in 20% (4/20; 95% CI, 5.7–43.7), with 16/20 patients completing neoadjuvant therapy [36]. Of the 40/50 participants who proceeded to surgery in the MATISSE trial, the use of two neoadjuvant doses of nivolumab or nivolumab/ipilimumab combination obtained a 40% and 53% mPR, respectively [37]. Further, two preliminary reports of trials employing a neoadjuvant plus adjuvant immunotherapy approach using only two doses of neoadjuvant immunotherapy reported pCR rates between 39 and 57% in 49 participants evaluable for response [38,39]. Altogether, the data so far argue that more rather than fewer doses of neoadjuvant ICB will increase the number of pCR cases observed, with four doses of neoadjuvant ICB achieving up to a 73% (95% CI 0.39–0.93) pCR rate, as observed in the DISCERN trial. Furthermore, given that high pCR rates can be achieved with neoadjuvant ICB alone, there is a strong impetus to investigate in future trials if careful patient selection may permit the omission of surgery, and if adjuvant immunotherapy after surgery is required. These questions are of particular importance given that the NADINA trial in stage III melanoma demonstrated that combination neoadjuvant ICB can achieve superior event-free survival (EFS) compared to an adjuvant approach, with an EFS rate of 83.7% (99.9% CI, 73.8–94.8) in the neoadjuvant ipilimumab and nivolumab group compared to 57.2% (99.9% CI, 45.1–72.7) observed in the adjuvant nivolumab group [16]. Lastly, given the lower response rate reported with a PD-L1 inhibitor-based neoadjuvant approach in CSCC, caution should be applied in extrapolating findings between PD-L1 inhibitor-based neoadjuvant therapy and PD-1 inhibitor-based therapy.

The optimal metabolic measure for assessing neoadjuvant response to ICB in CSCC requires ongoing refinement. In the DISCERN trial, although metabolic disease response assessment was in the same direction as pathological responses observed, both a PMR and CMR on pre-operative FDG-PET imaging preceded the pathological outcome of patients who obtained pCR, with 6/8 achieving a PMR and 2/8 a CMR. The impact of ICB on the tumour microenvironment is likely a metabolically active process, which could lead to the interpretation of residual avidity as a PMR rather than CMR, despite no viable disease remaining. Similarly, although the De-Squamate trial reported a high complete clinical response rate of 45% using CMR on FDG-PET and biopsies to confirm absence of disease, using CMR alone rather than PMR and CMR was likely to have missed identifying additional patients who could have avoided surgery [35]. The De-Squamate trial reported an additional 15% of pCR participants whose FDG-PET notably did not achieve a CMR prior to surgery. The data points to the limitation of FDG as a functional imaging tracer in neoadjuvant immunotherapy. Future trial designs incorporating metabolic response assessments to select patients to avoid surgery could consider immunotherapy alone for patients whose disease responds sufficiently to achieve a PMR on FDG-PET after four doses of neoadjuvant immunotherapy.

The iPERCIST and other response criteria require customisation for the neoadjuvant context. For example, the requirement for repeat staging after unconfirmed progressive metabolic disease (UPMD) does not facilitate rapid identification of patients who are not responding to immunotherapy. Metabolic response guidelines for CMR should also include consideration of physiological avidity in those with contralateral organs available for comparison. In two participants (008, 010), the pre-operative FDG avidity by SULpeak was symmetrical with the contralateral normal parotid, representing physiological uptake, although higher than the baseline measurement. Therefore, we propose that any definition of CMR should include both “complete resolution of FDG uptake within the target lesion or resolution of avidity symmetrical to a contralateral organ without disease (i.e., physiological avidity)”. Additionally, whether SULpeak is the optimal method of measurement and what the threshold percentage change is that reliably reflects a clinically meaningful response is still not clear [40,41,42,43,44]. For example, for patient 005, the pre-operative SULpeak change was more concordant with the depth of the pathological outcome of a pCR. That is, the SULpeak change of the target lesion was −44%, defining a PMR by iPERCIST criteria, whilst the SUVmax change was only −25%. However, the majority of SULpeak and SUVmax changes were similar in magnitude and direction.

Pathological response assessment is critical for the correct interpretation of the benefit of neoadjuvant immunotherapy administered prior to surgery, with it being reasonably presumed that the discernment between viable and non-viable CSCC would be simple for an expert pathologist. However, as reported in the supplementary data of the Phase II neoadjuvant cemiplimab trial, there was significant discordance of pathological response assessments between local and independent central review in 10/70 (14%) cases, whereby pCR cases were interpreted as non-responder cases and vice versa [19]. Of note, only the local pathology review was used by investigators to decide on whether further adjuvant therapy was required. This sobering discordance highlights the need for harmonisation of pathological assessment of ICB neoadjuvant response for CSCC, pertinent for trials with a primary endpoint of pCR. The United States Food and Drug Administration have issued a position statement on pCR as a potential surrogate endpoint to apply for accelerated approvals [45], but consensus guidelines for standardising tissue submission, assessment, and description of histopathological patterns of response have been developed for only a limited number of cancer types [31,46]. In CSCC, Ferrarotto et al. were the first to report pathological features of response to the PD-1 inhibitor cemiplimab, noting the presence of anucleate or non-viable keratin as a feature [15]. In their methods, the RVT percentage was calculated by “The relative percentages of tumour bed occupied by viable tumour, fibrosis, keratin, tumoural necrosis, and inflammatory infiltrate” for each sample, and the presence of a dense inflammatory infiltrate was observed. It was not described whether particular features of response were observed in primary cutaneous lesions or metastatic/nodal sites. Notably, in the DISCERN trial, few samples were noted to have tumour-infiltrating lymphocytes regardless of their location (epidermis versus nodal), and if present, these were considered minimal in density. In keratinising CSCC, the presence of anucleate keratin or “orthokeratin” is a key feature in the pathological assessment of neoadjuvant response to immunotherapy, similar to the presence of melanosis identified in regressed melanoma samples. We observed in one highly keratinised pNR patient case (009) three distinct areas: one orthokeratin-rich area associated with viable CSCC; a second area devoid of keratin and without viable CSCC but which had other pathological features of response; and a third orthokeratin-rich area that was not associated with viable CSCC or any other pathological features of response. We propose that the latter area should not be included in the tumour bed dimensions for the calculation of RVT. Keratin formation can arise in skin due to non-malignant processes such as ruptured epidermal cysts or inclusion cysts from a previous biopsy. A cavity with no obvious contents was also seen in this patient case, and we propose that these areas should only be included in the tumour bed dimensions for the calculation of RVT and considered evidence of tumour regression when cavitation is associated with pathological features of response to immunotherapy. At worst, when using these criteria, the RVT may then underestimate the response to neoadjuvant therapy.

## 5. Conclusions

In conclusion, the DISCERN neoadjuvant trial demonstrated that an excellent pCR rate of 73% (95% CI 0.39–0.93) can be achieved with the use of four doses of neoadjuvant cemiplimab. The trial highlights the limitations of imaging assessments, with most patients achieving only a PR by RECIST 1.1 and PMR by iPERCIST criteria, despite having a pCR.

Urgent work is required to customise imaging and metabolic response criteria to the neoadjuvant context, and it will be of critical importance to develop harmonised guidelines for the pathological assessment of neoadjuvant immunotherapy response for CSCC, with the criteria proposed to standardise the assessment of areas of acellular keratin. As multiple phase II/III neoadjuvant trials for CSCC (e.g., NCT06295809, NRG-HN014) investigate the role of neoadjuvant immunotherapy for locoregionally advanced CSCC, further advances may be facilitated by the identification of biomarkers to optimise patient selection for those with tumours that will respond, and by improved real-time assessment modalities, including imaging approaches that assist differentiation between pseudoprogression and progressive disease.

## Figures and Tables

**Table 1:** Baseline clinical demographics.

Characteristic		N (%)
Age (range) years		67 (55–83)
Sex Female: Male		1:10
Primary tumour site	Head and neck	9 (82)
	Hand	2 (18)
Overall Stage	III	3 (27)
	IV (M0)	8 (73)
Tumour stage	T1	1 (9)
	T2	2 (18)
	T3	4 (36)
	Tx	4 (36)
Nodal stage	N0	2 (18)
	N1	1 (9)
	N2a	1 (9)
	N2b	2 (18)
	N2c	2 (18)
	N3a	1 (9)
	N3b	2 (18)
ECOG performance status score	0	8 (73)
	1	3 (27)
Median duration of follow-up (IQR) months		10.2 (6.7–16.4)

**Table 2:** Summary of overall response.

Tumour Response	Number (%)	95% CI
**Pathological Response**		
Complete Response	8/11 (73)	0.39, 0.93
Non-Responder *	3/11 (27)	0.07, 0.61
**Imaging Response by RECIST 1.1**		
Partial response	8/11 (73)	0.39, 0.93
Progressive disease	3/11 (27)	0.07, 0.61

* All pathological non-responders had progressive disease with >50% residual viable tumour or new disease. See Table 4 for further details.

**Table 3:** Detailed response data according to RECIST 1.1, imRECIST, iPERCIST, and pathological response to neoadjuvant immunotherapy per participant.

ID	Timepoint	RECIST 1.1	imRECIST	iPERCIST	Pathological Response
**001**	**Day 75** Overall	PR (−30%)	PR (−30%)	PMR (−61%) Responder	pCR
**002**	**Day 75** Overall	PR (−58%)	PR (−58%)	* PMR (TL−100%, NTL PMR) Responder	pCR
**003**	**Day 75** Overall	PR (−31%)	PR (−31%)	CMR (−100%) Responder	pCR
**004**	**Day 75** Overall	PR (−42%)	PR (−42%)	PMR (−70%) Responder	pCR
**005**	**Day 75** Overall	PR (−29%)	PR (−29%)	PMR (−44%) Responder	pCR
**006**	**Day 75** Overall	PR (−43%)	PR (−43%)	PMR (TL−100%, NTL SMD) Responder	pCR
**008**	**Day 75** Overall	PR (−57%)	PR (−57%)	^#^ CMR (−74%) Responder	pCR
**010**	**Day 75** Overall	PR (−43%)	PR (−43%)	PMR (−63%) Responder	pCR
**009**	**^$^ Day 43** TL NTL New Overall	NE Non-CR/Non-PD No ^ PD	NE Non-CR/Non-PD No ^ PD	UPMD (+87%) UPMD No UPMD Non-responder	pNR ^&^ Primary 95% RVT, Epitrochlear node 60% RVT, Axillary nodal 90% RVT
**011**	**Day 43** TL NTL New Overall	PR (−45%) Non-CR/Non-PD Yes ^^ PD	PR (−45%) Non-CR/Non-PD Yes ^^ PD	SMD (+5%) UPMD Yes UPMD Non-responder	pNR ^&^ Primary 15% RVT, Axillary node >50% RVT, New level V nodal disease 100% viable
**012**	**^$^ Day 43** TL NTL New Overall	PD (+57%) NA No PD	PD (+57%) NA No PD	UPMD (+31%) NA No UPMD Non-responder	pNR/ progressive disease ^&^ Primary 100% viable

CR—complete response; CMR—complete metabolic response; NE—not evaluable (by imaging); Non-PR/Non-PD—non partial response/non progressive disease; NTL—non-target lesion; pCR—pathological complete response; PD—progressive disease; pNR—pathological non-responder; PMR—partial metabolic response; PR—partial response; SD—stable disease; SMD—stable metabolic disease; TL—target lesion; UPMD—unconfirmed progressive metabolic disease. * For iPERCIST, the non-target lesions were more FDG-avid and would have been selected as the target lesions for iPERCIST. However, the overall iPERCIST response is not altered. ^#^ Complete resolution of FDG uptake within measurable target lesion was achieved, which was less than mean liver activity and indistinguishable from surrounding background blood-pool levels, and disappearance of all other lesions to background blood-pool levels was observed. ^ PD status was allocated in retrospect. The patient had been admitted with concern of infection causing enlargement of cystic nodal target lesion and clinical deterioration resulting in pseudoprogression per RECIST 1.1 measurements. However, after treatment of infection and aspiration of the cystic node, multidisciplinary consensus was that disease progression could not be excluded, and the patient proceeded to surgery early, with pathological non-responder status confirmed. ^^ PD status was allocated in retrospect. At the time of the D43 scan, new non-RECIST measurable given the PR (−45%) achieved in the single target lesion. ^$^ Imaging timepoints were performed earlier than D43 due to clinical concern of progressive disease. ^&^ Anatomical lesions are not listed according to RECIST 1.1 target and non-target allocation.

**Table 4:** Pathological assessment of disease response in complete responder and non-responder cases.

PATHOLOGICAL COMPLETE RESPONDERS						
ID	Pathological Response (% RVT)		Largest Regression Bed Size		Closest Resection Margin (mm)	Extra-Nodal Extension?
	Primary	Nodal	Primary	Nodal	Regression Bed	Regression Bed
**001**	0	0	40	18	<0.1	Yes
**002**	NA	0	NA	16	0.2	No
**003**	0	NA	40	NA	Involved	NA
**004**	NA	0	NA	45	Involved	Yes
**005**	NA	0	NA	No *	NA *	No *
**006**	0	NA	NE ^#^	-	Involved	NA
**008**	0	0	22	11	Involved	No
**010**	NA	0	NA	42	Involved	No
**PATHOLOGICAL NON-RESPONDERS**						
**ID**	**Pathological response** **(% RVT)**		**Largest area of RVT/total tumour bed (mm)**		**Closest resection margin (mm)**	**Extra-nodal extension?**
	**Primary**	**Nodal**	**Primary**	**Nodal**	**Viable tumour**	**Viable** **tumour**
**009**	95%	Axilla 90% Epitrochlear 60%	43/50	Axilla 110/125 Epitrochlear 55/60	Primary involved Axilla 1 mm Epitrochlear involved	Present
**011**	15%	Axilla > 50% New level V 100%	55/55	New Level V 20/20	Margins clear	Present
**012**	100%	NA	43/43	NA	0.2	NA

NA—not applicable; NE—not evaluable; pNR—pathological non-responder; RVT—residual viable tumour. * No tumour bed was identified. That is, the entire specimen appeared normal. ^#^ Given the clinical complete response and metabolic complete response, a central wedge excision was performed with peripheral biopsies, with limited neck dissection. A total of 95% of the excisions had a regression bed identified.

**Table 5:** Pathological features of response to neoadjuvant immunotherapy according to density of feature. Bolded text within the table highlights the features of the pathological non-responder cases.

Patient ID	Regression Pattern	TIL	Tertiary Lymphoid Structure	Dense Plasma Cells	Cholesterol Clefts	Foamy Macrophages	Neo-Vascularisation	Proliferative Fibrosis	Granulomas	Giant Cells	Immune Exclusion
001	Mixed ^&^	No	Yes	No	No	No	No	Yes	Yes	Yes	No
002	Mixed	No	No	No	Yes	Yes	No	No	Yes	Yes	No
003	Fibrotic	No	Yes	No	No	No	No	Yes	Yes	Yes	No
004	Mixed	No	No	No	No	No	No	Yes	Yes	Yes	No
005	^#^ Normal	No	No	No	No	No	No	No	No	No	No
006	Mixed	No	Yes	No	No	No	No	No	Yes	Yes	No
008	Mixed	No	No	No	No	No	No	No	Yes	Yes	No
010	Mixed	No	Yes	No	Yes	Yes	No	Yes	Yes	Yes	No
**009**	**Inflamed**	**Yes**	**Yes**	**No**	**Yes**	**Yes**	**No**	**Yes**	**Yes**	**Yes**	**No**
**011**	**Mixed**	**Yes**	**No**	**No**	**No**	**Yes**	**No**	**Yes**	**Yes**	**Yes**	**No**
**012**	* None	**No**	**No**	**Yes**	**No**	**No**	**No**	**No**	**No**	**No**	**Yes**
											**Legend**
											Minimal
											Moderate
											Dense

^&^ Mixed—granulomatous inflammation and fibrosis with keratin. ^#^ No pathological features of tumour regression were seen with tissue considered completely within the normal range, and no viable tumour identified.* No regression was identified in this sample with 100% residual viable tumour and clinical progression of disease. TIL—tumour infiltrating lymphocytes.

**Table 6:** Changes to planned surgery and adjuvant radiation with the use of neoadjuvant immunotherapy for cutaneous squamous cell carcinoma.

ID	Planned Surgery	Actual Surgery	PORT Recommended
Complete Pathological Responders			
**001**	WLE of skin left occiput, bilateral SLND +/− local flap reconstruction + PORT	WLE of skin left occiput + rotational cervical flap + right posterolateral neck dissection	No
**002**	Parotidectomy and radical neck dissection +/− excision of facial nerve +/− accessory nerve sacrifice + PORT	Parotidectomy + SLND II/III	No
**003**	WLE + free flap +/− local flaps (full thickness cheek defect) + PORT	WLE DRAPE 1 (DRAPE 2 not required)	No
**004**	SLND + PORT	SLND IIa,b + Va	No
**005**	Subtotal parotidectomy +/− facial nerve sacrifice, right selective neck dissection (II/III/Va) + local flaps + PORT	Subtotal parotidectomy with facial nerve preservation + SLND I/II/III/upper V	No
**006**	Total lower lip resection (full thickness) + free flap reconstruction with bilateral (levels I–III) neck dissections + PORT	Wedge resection of the lip with SLND (level Ia, Ib bilateral)	No
**008**	WLE skin, parotidectomy +/− facial nerve dissection + SLND + PORT	WLE skin, parotidectomy + SLND (no flap required)	No
**010**	Parotidectomy +/- facial nerve resection + SLND + PORT	Superficial parotidectomy + SLND levels I/II/III/upper V	No
**Pathological Non-Responders**			
**009**	WLE, metastatectomy, ALND + free flap reconstruction of hand with tendon reconstruction + PORT	WLE with free flap reconstruction and tendon reconstruction, metastatectomy, ALND + flap reconstruction	Yes
**011**	WLE first web space + muscles +/− bone burring + regional flap + ALND (two levels) + PORT	Surgery 1: DRAPE 1 hand + ALND. Surgery 2: Re-excision. Surgery 3. Free flap reconstruction hand + right level V SLND	Yes
**012**	WLE (full thickness resection) lower lip with SLND, local flap reconstruction + PORT	WLE (full thickness lip) + free flap with bilateral I–III neck dissection	Yes

ALND—axillary lymph node dissection; DRAPE—Delayed Reconstruction After Pathological Evaluation; PORT—post-operative radiation therapy; SLND—selective neck dissection; WLE—wide local excision.

## Data Availability

Data are available upon reasonable request to the corresponding author, but only following the publication of the primary endpoint of the trial and completion of the study.

## Supplementary Materials

The following supporting information can be downloaded at: https://www.mdpi.com/article/10.3390/cancers17101727/s1, Table S1. Summary table of all adverse events reported as worst grade per event per patient; Table S2. Summary table of cemiplimab-related adverse events, including those possibly, probably, and definitely related, reported as worst grade per event per patient; Table S3. Summary of the intra-operative surgical assessments; Figure S1. Consolidated Standards of Reporting Trials (CONSORT) flow diagram; Figure S2. Representative images of the radiological response observed in Participant ID 006, in whom a pathological complete response was achieved; Figure S3. Representative images of progressive disease observed in Participant ID 011.

## Abbreviations

The following abbreviations are used in this manuscript:

AE	Adverse event.
CI	Confidence interval.
CSCC	Cutaneous squamous cell carcinoma.
CMR	Complete metabolic response.
CR	Complete response.
CT	Computerised tomography.
DISCERN	Deep sequencIng in cutaneous Squamous CEll caRciNomas.
DFS	Disease-free survival.
ECOG	Eastern Cooperative Oncology Group.
FDG	Fluorodeoxyglucose.
FDG-PET	Fluorodeoxyglucose-positron emission tomography.
HIV	Human immunodeficiency virus.
ICB	Immune checkpoint blockade.
IQR	Interquartile range.
irAE	Immune-related adverse event.
iPERCIST	Immune PET Response Criteria in Solid Tumours.
imRECIST	Immune-Modified Response Evaluation Criteria in Solid Tumours.
mPR	Major pathological response.
MRI	Magnetic resonance imaging.
NTL	Non-target lesion.
ORR	Overall response rate.
OS	Overall survival.
pCR	Pathological complete response.
PD	Progressive disease.
PD-1	Programmed Death-1.
PD-L1	Programmed Death Ligand-1.
PMR	Partial metabolic response.
pNR	Pathological non-responder.
PR	Partial response.
RECIST	Response Evaluation Criteria in Solid Tumours.
RVT	Residual Viable Tumour.
SAE	Serious adverse event.
SULpeak	Standardised uptake value peak.
SUVmax	Standardised uptake value max.
TIL	Tumour-infiltrating lymphocytes
TL	Target lesion.
UPMD	Unconfirmed progressive metabolic disease.
