# Supplementary material for: Radiological, Pathological, and Surgical Outcomes with Neoadjuvant Cemiplimab for Stage II–IV Cutaneous Squamous Cell Carcinoma in the Deep Sequencing in Cutaneous Squamous Cell Carcinomas (DISCERN) Trial
Source: Cancers (Basel). 2025 May 21;17(10):1727. doi: 10.3390/cancers17101727 (PMC12110075; doi:10.3390/cancers17101727)
Supplement: Supplementary file 1 [file cancers-17-01727-s001.zip › cancers-3588347-supplementary.pdf]

SUPPLEMENTARY MATERIALS

Supplementary Tables

Table S1. Summary table of all adverse events reported as worst grade per event per patient.

Table S2. Summary table of cemiplimab-related adverse events, including those possibly, probably, and definitely related, reported as worst grade per event per patient.

Table S3. Summary of the intra-operative surgical assessments.

Supplementary Figures

Figure S1. Consolidated Standards of Reporting Trials (CONSORT) flow diagram.

Figure S2. Representative images of the radiological response observed in Participant ID 006, in whom a pathological complete response was achieved.

Figure S3. Representative images of progressive disease observed in Participant ID 011.

TABLES

Supplementary Table S1. All adverse events reported as worst grade per each event per patient.

Adverse event	Cemiplimab 350mg Q3W (n=11)	
	Any grade	Grade ≥3
	No. of patients (%)	
**Any***	11 (100)	5 (45)
**Serious***	3 (27)	2 (18)
**Led to discontinuation**	0	0
**Led to death**	0	0
Alanine aminotransferase increased	2 (18)	
Anaemia	1 (9)	
Arthralgia	2 (18)*	
Aspartate aminotransferase increased	2 (18)	
Cardiac disorders other—exacerbation of CCF	1 (9)	
Confusion	1 (9)	
Constipation	2 (18)	
Diarrhoea	1 (9)	
Fall	1 (9)	
Fatigue	3 (27)*	
Haematoma		1 (9)
Hyperkalaemia		1 (9)*
Hypertension		1 (9)*
Hypophysitis		1 (9)
Infection and infestations other—axilla infection		1 (9)
Infusion-related reaction	2 (18)*	
Insomnia	1 (9)*	
Musculoskeletal and connective tissue disorder, other- tongue swelling from mucosectomy	1 (9)	
Nausea	3 (27)	
Nausea post-operative	1 (9)	
Pain	2 (18)*	
Pain—cancer	1 (9)	
Pain—operative	1 (9)	
Pain—post-operative	6 (54)	
Pruritus	1 (9)	
Rash maculo-papular	3 (27)	
Respiratory infection	1 (9)	
Skin and subcutaneous tissue disorder other - lichenoid skin reaction	1 (9)	
Skin and subcutaneous tissue disorders other - rash	1 (9)	
Sneezing	1 (9)	
Thrush	1 (9)	
Urinary tract infection		1 (9)
Urinary urgency	1 (9)	
Wound infection		1 (9)

*Multiple separate events that occurred in one individual are listed once

<u>Supplementary Table S2.</u> Cemiplimab-related adverse events, including those possibly, probably, and definitely related, reported as worst grade per event per patient.

**Adverse event**	**Cemiplimab 350mg Q3W (n=11)**	
	**Any grade**	**Grade ≥3**
	**No. of patients (%)**	
**Any***	10 (91)	1 (9)
**Serious**	1 (9)	1 (9)
**Led to discontinuation**	0	0
**Led to death**	0	0
Alanine aminotransferase increased	2 (18)	
Arthralgia	2 (18)*	
Aspartate aminotransferase increased	2 (18)	
Diarrhoea	1 (9)	
Fatigue	3 (27)*	
Hypophysitis		1 (9)
Infusion-related reaction	2 (18)*	
Pruritus	1 (9)	
Rash maculo-papular	3 (27)	
Skin and subcutaneous tissue disorder other- lichenoid skin reaction	1 (9)	
Skin and subcutaneous tissue disorders other- rash	1 (9)	
Sneezing	1 (9)	

* Multiple separate events that occurred for an individual participant are listed once

Supplementary Table S3. Summary of the intra-operative surgical assessments.

Question	Pathological Responder versus Non-Responder	Yes (%)
**1. Based on the pre-operative examination findings alone, did the surgeon anticipate residual macroscopic disease?**	**pCR**	
	Primary	1/8 (12.5)
	Lymph nodes	1/8 (12.5)
	**pNR**	
	Primary	3/3 (100.0)
	Lymph nodes	2/3 (66.6)
**2. Were surgical margins changed due to intra-operative findings?**	**pCR**	1/8 (12.5)
	**pNR**	1/3 (33.3)
**3. Did the intra-operative findings change the extent of planned surgery?**	**pCR**	1/8 (12.5)
	**pNR**	0/3 (0.0)
**4. Intra-operatively, did the surgeon feel that there was residual macroscopic disease?**	**pCR**	
	Primary	3/8 (37.5)
	Lymph nodes	3/8 (37.5)
	**pNR**	
	Primary	1/3 (33.3)
	Lymph nodes	2/3 (33.3)
**5. Surgical assessment of the feel of the tissues and surgical plane according to the following grading;**	**Primary Site**	
	**pCR**	
	0	1/8 (12.5)
	1	3/8 (37.5)
0. No change to tissues	2	2/8 (25.0)
1. Mild fibrosis (no substantial impact on conduct of surgical resection)	3	0 (0.0)
	4	0 (0.0)
2. Moderate fibrosis (requires increased effort and dissection during resection, but otherwise does not severely impact the conduct of the surgery)	NA	2/8 (25.0)
	**pNR**	
	0	2/3 (66.6)
	1	1/3 (33.3)
3. Severe fibrosis (substantially impacts the conduct of the operation by increasing the duration of or blood loss during the surgery, or requires a significant alteration in technique)	2-4	0 (0.0)
	**Lymph Nodes**	
	**pCR**	
	0	0 (0.0)
4. Severe fibrosis resulting in unresectability	1	3/8 (37.5)
	2	4/8 (50.0)
	3	0 (0.0)
	4	0 (0.0)
	NA	1/8 (12.5)
	**pNR**	
	0	2/3 (66.6)
	1	1/3 (33.3)
	2-4	0 (0.0)

**6. Operative Time in median minutes (range)**	**pCR**<br><br>**pNR**	211<br>(30-309)<br>240<br>(202-420)

NA—not applicable; pCR—pathological complete response; pNR—pathological non-responder

FIGURES

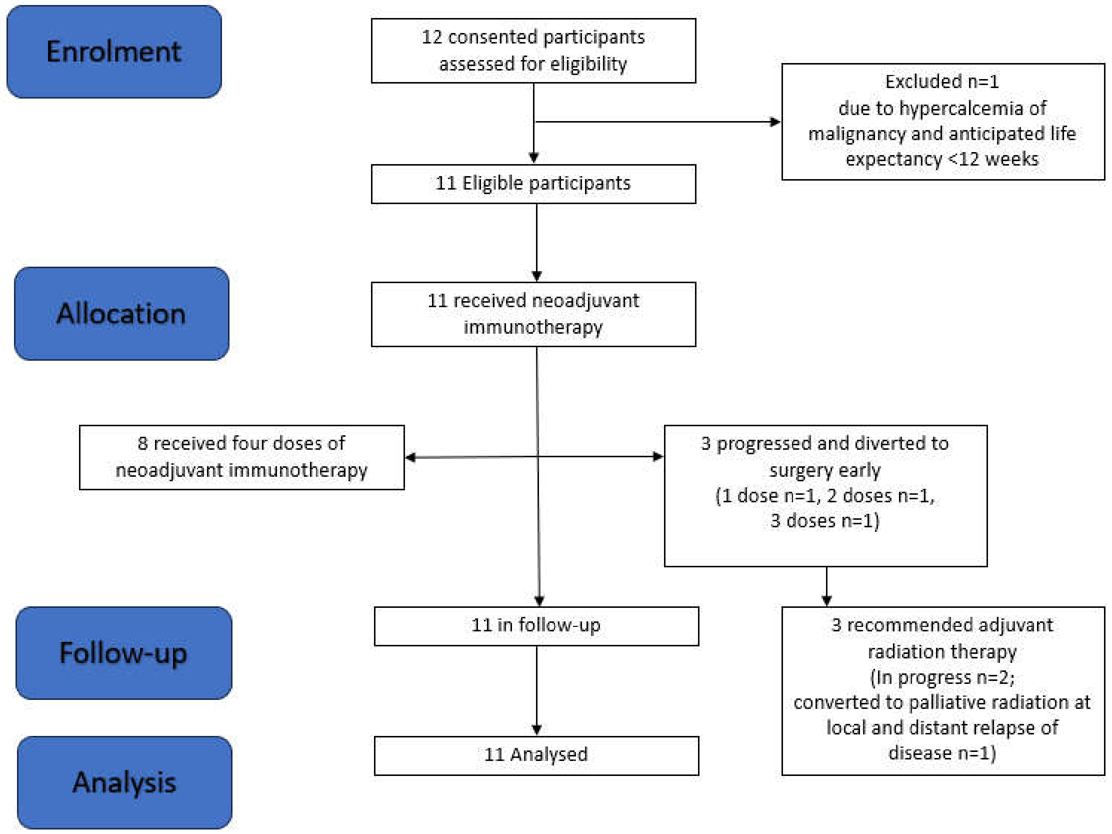

Supplementary Figure S1. Consolidated Standards of Reporting Trials (CONSORT) flow diagram.

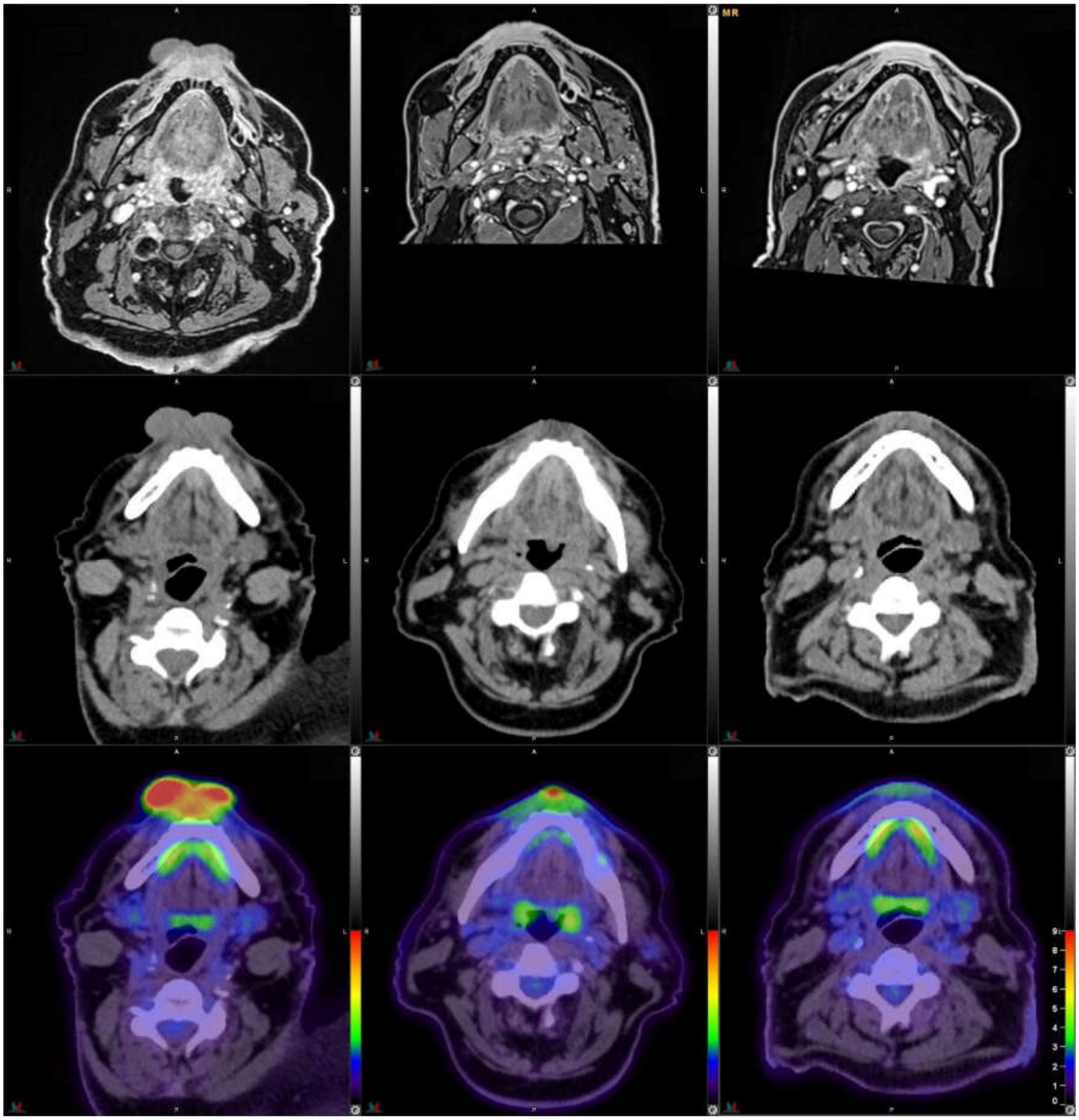

Supplementary Figure S2. Representative images of the radiological response observed in Participant 006, in whom a pathological complete response was achieved. Top row: Intravenous contrast-enhanced T1-weighted axial MRI images pre-treatment, post 2 doses of cemiplimab (~Day 43), and post 4 doses of cemiplimab (left to right) showing involution of the large exophytic lower lip lesion. Middle and bottom rows: CT and fused axial FDG PET-CT images. The left images demonstrate the large lower lip CSCC pre-treatment, with intense FDG avidity. The middle images demonstrate a partial metabolic response and significant structural improvement post 2 doses of cemiplimab, and the right images demonstrate a complete metabolic response post 4 doses of cemiplimab.

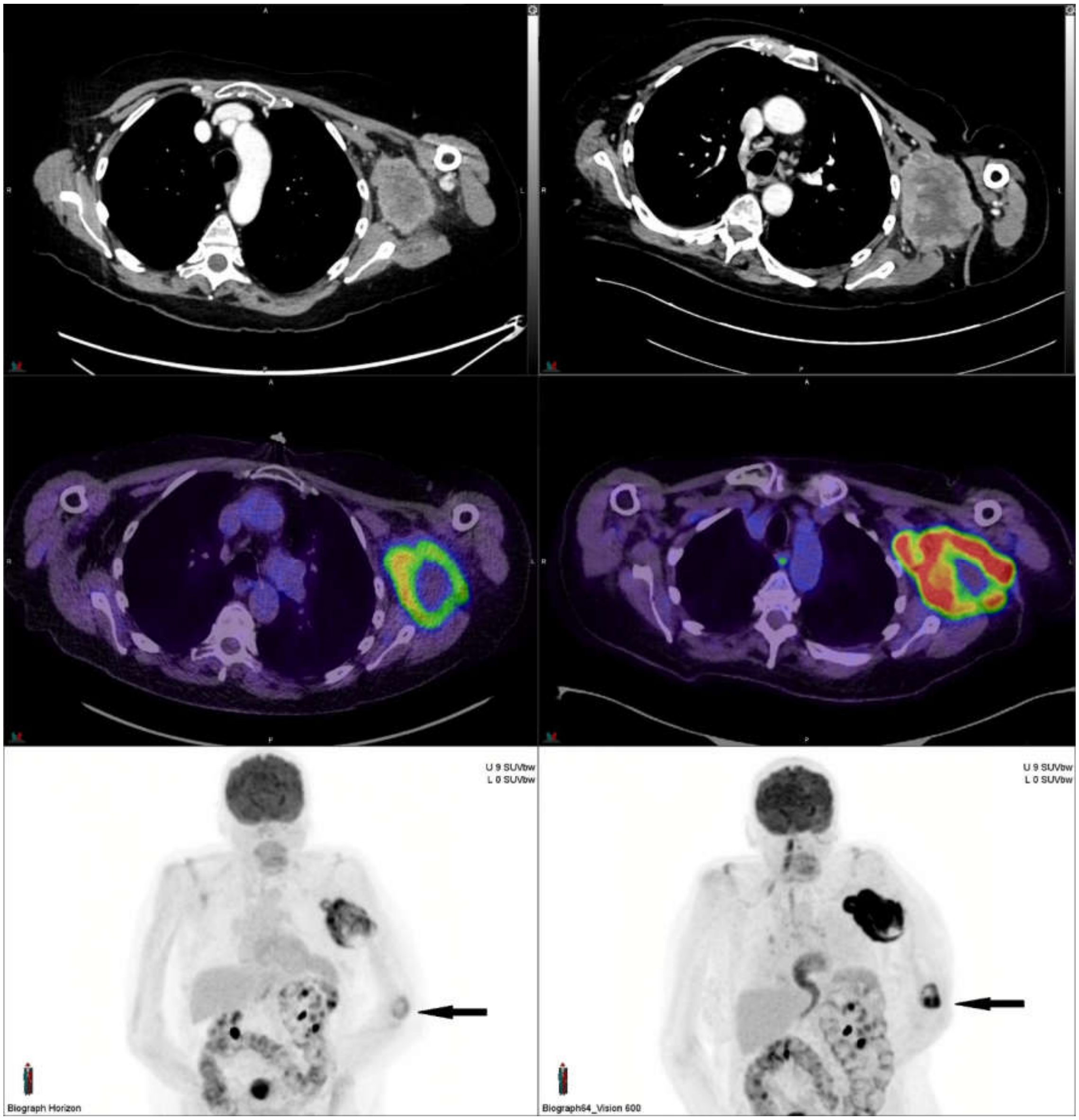

Supplementary Figure S3. Representative images of progressive disease observed in Participant ID 011. Intravenous contrast enhanced axial CT images (top row), fused axial FDG PET-CT images (middle row) and PET maximum intensity projection (MIP) images bottom row, demonstrating a large centrally necrotic left axillary mass pre-treatment (left) with an increase in size and avidity observed post 2 doses of cemiplimab (right) consistent with disease progression/non-response. Arrows on the MIP images demonstrate metabolic progression of an additional left epitrochlear lesion.
